# ASO Author Reflections: APRI + ALBI: A Novel Tool for Estimating Chemotherapy-Associated Liver Injury in Patients with Colorectal Cancer Liver Metastasis Undergoing Liver Resection

**DOI:** 10.1245/s10434-019-07395-8

**Published:** 2019-04-29

**Authors:** D. Pereyra, P. Starlinger

**Affiliations:** Department of Surgery, General Hospital, Medical University of Vienna, Vienna, Austria

## Past

Within the past decade, the management of patients treated for colorectal cancer liver metastasis changed rapidly and evolved to be one of the most prominent fields within surgical oncology. Perioperative chemotherapy improves the outcome of both resectable and initially unresectable colorectal cancer liver metastasis.^1^ Despite the benefit in terms of oncologic outcome, chemotherapy-associated liver injury is frequently observed in these patients and shown to have a significant negative impact on the postoperative clinical outcome after liver resection.^2^^,^^3^ This is of specific relevance because postoperative morbidity significantly affects the overall oncologic outcome of colorectal cancer liver metastasis. Yet, to date, no valid tool exists to assess chemotherapy-associated liver injury. Therefore, preoperative risk stratification after neoadjuvant chemotherapy remains a difficult task for clinicians worldwide.

## Present

This study aimed to define a tool to assess chemotherapy-associated liver injury after neoadjuvant chemotherapy.^4^ The study focused particularly on patients with colorectal cancer liver metastasis undergoing liver resection. The marker chosen was to be based on routine laboratory data and easily applicable within the routine clinical setting. Furthermore, the evaluated marker was not only to be associated with chemotherapy-associated liver damage, but also to be a sensitive predictor of deteriorated postoperative outcome. In this context, APRI + ALBI, a composite of aspartate aminotransferase, platelet count, albumin, and bilirubin, was found to be increased in patients displaying chemotherapy-associated liver injury. Moreover, an increase after completion of neoadjuvant chemotherapy was observed. Ultimately, APRI + ALBI was found to facilitate preoperative risk stratification in patients after neoadjuvant chemotherapy via identification of patients more likely to experience postoperative liver dysfunction and morbidity.

## Future

In summary, APRI + ALBI allows estimation of chemotherapy-associated liver damage over time. This represents a unique property because to date no noninvasive tool exists to monitor liver damage after neoadjuvant chemotherapy. Furthermore, the findings showed that APRI + ALBI dynamically monitors liver damage because it declined within the chemotherapy-free interval before the operation, presumably reflecting gradual liver function recovery. In addition, given the association with postoperative outcome, APRI + ALBI can be used to identify patients experiencing clinically relevant chemotherapy-associated liver injury. Ideally, this marker, which was not only established but also validated in a multicenter fashion, will be used as a tool for personalized scheduling of liver resection after neoadjuvant chemotherapy. Patients exceeding the proposed cutoff before the operation might benefit from postponement of liver resection until liver function has further improved. This might eventually lead to a reduction in postoperative complications and hence improvement of the overall outcome for this specific patient cohort.
